# Practical Experience of Endoscope Reprocessing and Working-Platform Disinfection in COVID-19 Patients: A Report from Guangdong China during the Pandemic

**DOI:** 10.1155/2020/9869742

**Published:** 2020-12-31

**Authors:** Xianqi Lin, Zhenyi Zhang, Minzhao Gao, Zhenling Zhang, Zhidong Lin, Siwen Huang, Jiangnan Ren, Meijuan Luo, Xiaoqing Xu, Guilan Chen, Hongjun Chen, Renxu Lai, Xiaochun Wu, Lihua Zhang, Xiaofeng Li

**Affiliations:** ^1^Department of Gastroenterology, The Fifth Affiliated Hospital, Sun Yat-sen University, Zhuhai, 519000 Guangdong, China; ^2^Department of Hospital Infection Management, The Fifth Affiliated Hospital, Sun Yat-sen University, Zhuhai, 519000 Guangdong, China

## Abstract

**Background:**

No consensus exists regarding which procedures should be performed to disinfect endoscopes and working platforms after COVID-19 patients have undergone endoscopy.

**Methods:**

We analyzed the disinfection quality of endoscopes and working platforms after 11 COVID-19 patients had undergone endoscopy.

**Conclusions:**

For endoscopic preprocessing at the bedside, a key disinfection step is using a multienzyme stock solution. The nucleic acid tests for endoscopists, washers, endoscopes, and working platforms were all negative. Based on our experience with the 11 COVID-19 patients who had undergone endoscopy, we provide an endoscopic reprocessing method for the bedside endoscopic diagnosis and treatment of COVID-19 patients for reference.

## 1. Introduction

Severe acute respiratory syndrome coronavirus 2 (SARS-CoV-2) produces the disease called coronavirus disease 2019 (COVID-19), which has spread worldwide as a transmissible respiratory disease. The most common symptoms of COVID-19 at the onset of illness are fever, cough, fatigue, myalgia, dyspnea, and GI symptoms [1–5]. The virus can be transmitted by contact, droplets, and aerosols and has been isolated from gastrointestinal secretions and feces [6–8]. Therefore, to avoid the spread of the virus, the cleaning and disinfection of endoscopes and working platforms are essential.

Our hospital, The Fifth Affiliated Hospital, Sun Yat-sen University, is a designated health care provider for COVID-19 in Zhuhai, where 103 cases of COVID-19 were treated. From January 17 to February 15, 2020, 11 patients had undergone bedside endoscopy. We focused on infection control and occupational protection against COVID-19 and summarized our experience with 11 COVID-19 patients who had undergone gastrointestinal endoscopy.

## 2. Methods

### 2.1. Health Care Professional Protection

According to the three-level protection requirements [9], in the buffer area, before entering the negative pressure ward [10], endoscopists and nurses should dress in specialized personal protection equipment (PPE), including medical protective clothing (disposable), impermeable isolation gowns, medical protective caps, waterproof boot covers, medical shoe covers, N95 medical protective masks, full-face respiratory protectors or positive pressure headgear, and double-layered gloves. Endoscope reprocessing operators should wear medical protective clothing (disposable), impermeable isolation gowns, medical protective caps, waterproof boot covers, medical shoe covers, N95 medical protective masks, protective face shields, and double-layered gloves in the buffer area before entering the endoscope reprocessing area. The health care professionals should remove their PPE in the buffer area before returning to clean working areas.

### 2.2. Endoscope Reprocessing

#### 2.2.1. Bedside Preprocessing

According to WS507-2016 [11], after endoscopy of the COVID-19 patients, bedside preprocessing of the endoscope was performed. After the completion by the endoscopists, the preprocessing operator immediately spirally scrubbed the outer surface of the endoscope operation part and outer surface of the insertion part with gauze containing multienzyme stock solution to avoid possible hazards of saliva adhering to the endoscope surface. The head end of the endoscope was immersed in the multienzyme stock solution, with repeated injection of gas/water until the tube was free from dirt under visual inspection. Next, 200 ml of the multienzyme stock solution was drawn into and maintained in the endoscope lumen for 2 minutes. To prevent protein solidification and crystallization caused by contact of the mucus on the endoscope lumen with the disinfectant, reduce the virus residue, and prevent the blocking of the endoscope lumen, we increased the concentration of the multienzyme and used the multienzyme stock solution directly. After removing the endoscope, the waterproof cap with the endoscope water injection bottle placed in a double-deck yellow medical bag (gooseneck type) was installed and labeled with the patient information and the word “COVID-19.” The medical bag was then sealed and transported through a special channel to the designated endoscope reprocessing room.

#### 2.2.2. Endoscopic Cleaning

Endoscope reprocessing operators performed three-level protection as required and wore a face shield to prevent the generation of aerosol during the cleaning process. The endoscope was completely immersed in the tank of multienzyme solution, the endoscope body was repeatedly scrubbed with a gauze block, and the head end and operation part were mainly scrubbed. Next, the endoscope biopsy pipeline was washed with a pressure water gun. The opening valve, water supply and air supply button, and suction button were cleaned and soaked in the multienzyme cleaning solution. A disposable endoscope cleaning brush was used to scrub the endoscopic biopsy pipe and suction pipe. When brushing, the brush head at both ends was visible and the dirt was cleaned on the brush head. Brushing was performed repeatedly until no debris and tissues were visible to the naked eye (the brush was rotated when entering and withdrawing). The brush head was washed again, rinsed with a pressure water gun, and blow dried with a pressure air gun (the level was not higher than the groove surface to minimize the generation of aerosols). A dedicated button for endoscope cleaning was installed, the injection port in the automatic perfusion device was connected to the endoscope perfusion connection tube, and the “start” button of the full-channel perfusion device was touched to fully brush and clean each lumen of the endoscope.

#### 2.2.3. Endoscopic Rinse

The cleaned endoscope, its components, and accessories were placed in the rinsing tank with flowing water. The full-tube perfusion device was turned on for full perfusion cleaning, and each pipe of the endoscope was thoroughly rinsed for at least 10 seconds, followed by rinsing the outer surface, parts, and accessories of the endoscope with flowing water. A pressure air gun was used to remove the moisture in the pipe and blow dry. Finally, the outer surface, parts, and accessories of the endoscope were dried.

#### 2.2.4. Endoscope Disinfection

The endoscope was completely immersed in the 0.55% orthophthalaldehyde (OPA) disinfectant for 5 minutes, and the endoscope pipe was filled with disinfectant using a fully automatic perfusion device or syringe, which disinfects for the prescribed time.

#### 2.2.5. Final Rinse

Hands were washed with flowing water, and double gloves were replaced. Next, the perfusion connector was removed, and then, the endoscope was removed from the disinfection tank and placed in the final rinse tank. The full-tube perfusion device (or pressure air gun, syringe) was connected to inflate various pipelines and discharge the disinfectant solution in the pipeline. Additionally, the full-tube perfusion device (or pressure gun, syringe) repeatedly rinsed each pipe of the endoscope with pure water for at least 2 minutes and fully rinsed the outer surface of the endoscope and accessories.

#### 2.2.6. Drying

The endoscope subjected to terminal rinse was placed on the drying table where disposable sterile treatment towels were placed. The full-tube perfusion device (or pressure air gun, syringe) was turned on, and all the tubes were flushed with 75% ethanol until the insertion end of the endoscope was full, and then, the endoscope lumen was filled with 75% ethanol and maintained for at least 5 minutes. A pressure air gun (filtered clean compressed air) was used to dry the surface of the endoscope and each pipeline, and the outer surface of the endoscope components and accessories was dried with sterile gauze. The dedicated endoscope cleaning button and perfusion connection tube were disassembled.

#### 2.2.7. Sterilization

The dried endoscopes, buttons, water injection bottles, and other related accessories were sent to the central supply room for repackaging and packaging, and then sterilization with ethylene oxide gas (ethylene oxide sterilization machine from 3M: sterilization temperature, 55°C; sterilization time, 1 hour; concentration, ~600 mg; entire treatment time, 12 hours).

### 2.3. Final Treatment of Inspection Room and Decontamination Room

#### 2.3.1. Diagnostic Equipment Such as Endoscope Host and Computer with Graphic System

Seventy-five percent alcohol was used to wipe and sterilize the surfaces of endoscope trolleys, endoscope host panels, endoscope display screens, computer display screens, computer mainframes, and computer mice for at least 30 minutes. The room was sterilized by ultraviolet radiation for 1 hour. After the final treatment at the bedside, the relevant equipment was pushed to the designated area for standby.

#### 2.3.2. Inspection Room

The walls and floors were wiped with 2000 mg/L of chlorine-containing disinfectant. After more than 30 minutes, the room was sterilized with ultraviolet light for 1 hour.

#### 2.3.3. Washing Room

The cleaning tank and full-tube perfusion device were continuously perfused with 2000 ppm of effective chlorine solution for 30 minutes, followed by the addition of fresh water for 5 minutes and then drying. Next, 2000 mg/L of effective chlorine solution was used to wipe and disinfect from the reverse cleaning direction of the rinse tank. After 30 minutes, the surface was wiped with clean water. Finally, the tank was washed, and the drying table was wiped with disinfectant wipes. The disinfection room was closed and exposed to ultraviolet light for 1 hour, and the walls and floors were wiped with 2000 mg/L of chlorine-containing disinfectant and allowed to stand for more than 30 minutes.

### 2.4. Final Treatment and Detection of Nucleic Acid on the Surface of the Endoscope before the Withdrawal of Medical Staff and Equipment

#### 2.4.1. Nucleic Acid Detection and Medical Observation of Medical Staff

The medical staff, including doctors, nurses/technicians, and sterilizers, were subjected to the necessary 14-day isolation medical observations, and nucleic acid was tested twice in parallel with 24-hour intervals, and they returned to work only when the result is negative.

#### 2.4.2. End-to-End Reprocessing before the Withdrawal of Endoscope-Related Equipment

After all the patients with COVID-19 were discharged from the hospital, the endoscope operation department and body, endoscope lumen, endoscope host, computer equipment, and treatment trolley were completely reprocessed (Table 1). Among them, the full-time engineer of the manufacturer mainly disassembled the endoscope host and the internal water/air pipe of the water pump/air pump and the air pump fan in the endoscope host were cleaned of the attached dust with a blower. After disassembling the endoscope, the cracks at the internal angles were cleaned and wiped with alcohol to prevent the entrapment of viruses. After cleaning all the equipment, fumigation treatment was carried out in a closed room with a hydrogen peroxide air sterilizer. Each surface of the above equipment was sampled twice to detect nucleic acids, and the results were all negative.

## 3. Discussion

The digestive tract is one of the target organs of SARS-CoV-2 [6]. Therefore, many studies have suggested that 3%–65.9% of patients with COVID-19 have gastrointestinal symptoms [1, 12]. During the examination, aerosols were easily generated; therefore, the risk of infection of medical staff for endoscopy is extremely high.

In response to this special situation, we propose the following: (1) epidemic emergency management: establish a specialty epidemic emergency team, comprising 5 senior doctors, 5 senior nurses, and 2 experienced disinfectors, to form a 12-member emergency team. (2) Team member management: to ensure the safety of patients and medical staff, the team members are given relevant protection and intensive training on the diagnosis process and multiple drills, including hand washing and putting on and taking off protective clothing, the endoscopy examination process, the endoscope reprocess, and other processes. All the staff must pass the evaluation before they can work, and the inspection personnel was relatively fixed to ensure proficiency in the diagnosis, treatment, and endoscope reprocessing. (3) Management of diagnostic equipment: as the only designated hospital in the municipal level, we prepared 2 endoscope mainframes, 12 endoscopes including gastrointestinal endoscopes, and 2 sets of endoscopy computer systems for the COVID-19 patients. The special endoscope host machine used by patients with COVID-19 is a dedicated machine, and each patient used the same endoscope. After all the endoscopes have been used, they were decontaminated and sterilized according to the process. The department has temporarily sealed the equipment and confirmed that the nucleic acid test was negative before it can be used again. (4) Bedside preprocessing of the endoscope was one of the most critical steps. After the bedside examination, the endoscope was transfer to the reprocessing room needed a long time. If there was no preprocessing with multienzyme stock solution, during the transfer, the endoscope body and lumen could easily result in protein coagulation and crystallization, leading to the blockage of the endoscope lumen and retention of the virus, which can cause infection. (5) After the inspection, in addition to disinfecting the surface of the endoscope host and instrument of the graphic system, the internal water/air pipe and air pump of the water pump/air pump of the endoscope host were treated to avoid residual dust caused by the virus. After disassembly from the endoscope host, the circuit board of the host and gap between the angles in the host were cleaned and disinfected, and then fumigated with hydrogen peroxide air to ensure further virus kill. After this treatment, we sampled everywhere and tested twice for nucleic acids.

We performed detailed gastroscopy for 11 COVID-19 patients. In addition to zero-distance contact with patients, digestive tract secretions also directly contaminate the gastroscope, accessories, and work area. Due to the spreading characteristics of the virus, a series of disinfection measures were further taken, and strict implementation and inspection were carried out. No infections by medical staff occurred, and no viruses attached to the endoscope-related host and accessories, thereby reaching the standard of disinfection. We have accumulated experience in further effectively preventing the spread and infection of SARS-CoV-2.

## 4. Conclusions

The reprocessing of gastroenteroscopy in patients with SARS-CoV-2 infection was described. We found that bedside preprocessing with multienzyme solution is a key step. According to our procedures, the nucleic acid tests of endoscopists, decontamination personnel, endoscopes, and working platforms were all negative. Whether our experience is entirely applicable to the reprocessing of gastrointestinal endoscopy in patients with SARS-CoV-2 infection must be further explored.

## Figures and Tables

**Table 1:** Nucleic acid sampling results before the evacuation of endoscopic diagnosis and treatment equipment for patients with COVID-19.

	Sample	Times	Results
Endoscope	Endoscope lumen	2	-
	Operation section+endoscope body surface	2	-
Trolley	Trolley surface	2	-
	Wheels	2	-
Mainframe	Display button	2	-
	Display surface	2	-
	Panel	2	-
	Endoscopic Bayonet	2	-
	Water and gas interface	2	—
	White balance calibration device	2	-
	Keyboard	2	-
	Keyboard holder	2	-
	Heat sink	2	-
Computer of graphic system	Button and panel	2	-
	Heat sink	2	-
	Data cable connection port	2	-
	Display button	2	-
	Display screen	2	-
Mainframe disassembly	The inner water and gas pipe of the main pump	2	-
	Circuit board	2	-
	Angle inside the mainframe	2	-

## Data Availability

The research data are available on request after institutional review board approval by contacting the corresponding author.
